# Improving the learning of chemical-protein interactions from literature using transfer learning and specialized word embeddings

**DOI:** 10.1093/database/bay066

**Published:** 2018-07-12

**Authors:** P Corbett, J Boyle

**Affiliations:** Data Science Group, Technology Department, The Royal Society of Chemistry, Thomas Graham House (290), Science Park, Milton Road, Cambridge CB4 0WF, UK

## Abstract

In this paper, we explore the application of artificial neural network (‘deep learning’) methods to the problem of detecting chemical-protein interactions in PubMed abstracts. We present here a system using multiple Long Short Term Memory layers to analyse candidate interactions, to determine whether there is a relation and which type. A particular feature of our system is the use of unlabelled data, both to pre-train word embeddings and also pre-train LSTM layers in the neural network. On the BioCreative VI CHEMPROT test corpus, our system achieves an *F* score of 61.51% (56.10% precision, 67.84% recall).

## Introduction

The BioCreative VI CHEMPROT task (1) concerns the detection of mentions of interactions between chemical compounds/drugs and genes/proteins. Prior to BioCreative VI there has been a limited amount of work on chemical-protein interactions, which has been reviewed in depth by Krallinger *et al.* 2017 (2). An early proposal was published by Craven and Kumelien in 1999 (3), and an early working system was published by Rindflesch *et al.* in 2000 (4). Recent examples (5–8) have used a variety of techniques, ranging from co-occurrence based approaches to parser-based systems and applied them to the generation of databases.

The neural network techniques known as ‘deep learning’ (9) have recently attracted a lot of interest in natural language processing. For example, in the recent BioCreative V.5 evaluation of chemical named entity recognition (NER) systems (10), the three highest-scoring systems all used deep learning approaches––in particular the recurrent neural network (RNN) technique known as Long Short Term Memory (LSTM; 11). RNNs have been applied to relationship extraction tasks––for example, Vu *et al.* (12) have applied connectionist RNNs to the SemEval-2010 relation classification task, Xiao and Lu (13) have applied LSTMs to the same task and Kavuluru *et al.* (14) have applied LSTMs to the task of detecting drug-drug interactions.

The CHEMPROT corpus consists of PubMed abstracts manually annotated with chemical compound mentions, gene/protein mentions and chemical compound-protein relations. Each relation annotation has one chemical compound mention, one gene/protein mention and a relationship type. There are 22 relationship types, collected into 10 groups, of which five groups are used in the CHEMPROT task (relations from the other five groups are discarded). The five relation groups are upregulator/activator (CPR: 3), downregulator/inhibitor (CPR: 4), agonist (CPR: 5), antagonist (CPR: 6) and substrate/product (CPR: 9). The annotated abstracts are provided in three groups––1020 training abstracts, 612 development abstracts and 800 test abstracts. During the BioCreative VI challenge, the test abstracts were mixed in with a further 2599 abstracts annotated for entities but not relationships, to ensure that participating systems did scale and to avoid manual corrections of the results.

We decided to examine the use of deep learning methods, in particular LSTM layers. One advantage of these deep learning methods is that they provide methods for exploiting unlabelled data, by means of transfer learning––training a neural network on some task with just the use of an unlabelled corpus and re-using trained components from that network in the task of interest.

An early example of transfer learning in neural networks for natural language processing was Collobert *et al.*’s SENNA system (15), which performed part-of-speech (POS) tagging, chunking, NER and semantic role labelling (SRL). To boost performance, the system was trained on a language modelling task on a large (∼852 million words) unlabelled data set. A language model is defined as a probability distribution over sequences of words. Rather than directly attempting to estimate such a probability distribution, Collobert *et al.* proposed a pairwise ranking approach, seeking a function that returns a higher score when presented with a legal phrase -i.e. one drawn from the corpus––rather than with an incorrect one–i.e. one not drawn from the corpus, but prepared by replacing one word with a different word. These artificial incorrect phrases have come to be known as ‘negative samples’, although that term was not used in the early literature on the technique.

The lowest layer of the Collobert *et al.* network is an ‘embedding’ layer, where each word is represented as a lookup table feature vector, randomly initialized and trained by backpropagation. The feature vector for each word is known as its embedding. Training the system on the language modelling task trained the embeddings. It was found that once trained by this method, the embedding for a given word was likely to be similar to the embeddings for similar words––for example, the nearest neighbours (by Euclidean distance) to ‘France’ were ‘Austria’ and ‘Belgium’. The trained embedding layer that had been generated by that procedure was then re-used in networks to perform other tasks, e.g. POS tagging and it was found that performance was boosted over and above using an embedding layer than had not been pre-trained in such a way.

Collobert *et al.* obtained further improvements by multi-task learning––jointly training the system on the POS, chunking, NER and SRL tasks was found to improve performance on three of the four tasks over just training the network for the task in question. Further knowledge had been transferred from task to task.

Techniques for producing embeddings were further developed by Mikolov *et al.* (16), producing the word2vec embeddings, and Pennington *et al.* (17), producing the GloVe embeddings. One feature of these methods is the use of a ‘skip-gram’ task, predicting a word’s context given the word itself. In GloVe, backpropagation is not used––instead an efficient procedure was designed specifically for the task. Pennington *et al.* also made sets of embeddings trained using GloVe available online. Some were trained on a combination of Wikipedia and the Gigaword corpus. Wikipedia contains a broad selection of content, including much of relevance to protein-chemical interactions. However, it is possible that embeddings trained on a more specialized corpus could offer better results; in this paper, we decided to investigate this.

These embedding methods enable transfer learning for the first layers of networks, but the features in subsequent layers may also be transferrable. Yosinski *et al.* (18) investigated transferability in image processing tasks, showing that transferring multiple layers of a network can further increase performance over and above the increase from transferring the first layer. In SENNA, Collobert *et al.* were able to show a benefit from jointly training a system to perform POS, chunking, NER and SRL tasks over individually training systems to perform each task. Given these results, we decided to investigate transfer learning beyond word embeddings, using a language modelling task to pre-train LSTM layers for use in a network to recognize chemical-protein interactions.

## Materials and methods

### Resources

We used various external components in our system. The software components include the deep learning toolkit keras–using tensorflow as the back end, python 3.6.1 and the tokenizer chemtok, as implemented in the chemical NER system ChemListem (19) (Chollet, F. (20^15^) “Keras” https://github.com/fchollet/keras.).

To prepare specialized pre-trained word embeddings, we used the Stanford GloVe software (17; as checked out from version control 3 July 2017). GloVe offers both some publicly available pre-trained embeddings, and also the software to compile your own––in previous work (19) we had success with the public embeddings, whereas here we compiled our own. To create these embeddings we prepared three corpora––the full texts of patents, consisting of patents with CPC codes A61K31 or A61P, from 2006 to November 2016, the full text of chemistry journal papers, consisting of papers published by the Royal Society of Chemistry from 2000 to end of 2016, and the titles and abstracts from PubMed records from 1809 to the end of 2015. As a comparison, we also tried using the public emdeddings, described on the GloVe web site as ‘Wikipedia 2014 + Gigaword 5 (6B tokens, 400 K vocab, uncased, 50d, 100d, 200d and 300d vectors, 822 MB download)’––here, we used the 300 dimensional vectors.

To make the initial embeddings, we extracted the text from the documents in the three corpora, tokenized it, outputting whitespace-separated tokens as one large text file. The contents of one document were separated from the next using lines consisting of a specialized repeated token. This file contained 6.7 billion tokens, about the same size as the corpus for the public embeddings.

We used the GloVe software to extract 300-dimensional vectors from the file, using the window size = 15, xmax = 100.

These specialized embeddings go some way towards ameliorating the out-of-vocab issue common in domains such as biomedical text, but not completely. In the training corpus, we were able to detect 21 378 distinct tokens that occurred twice or more (our system treated tokens that occurred only once in the training corpus as out-of-vocab), of which 11 872 occurred in the specialized embeddings and 6933 occurred in the public embeddings. Embeddings for tokens in the training corpus but not in the GloVe embeddings were initialized with zeroes.

We also prepared a file for transfer learning, taking the titles and abstracts from PubMed as mentioned above. The file consisted of one paragraph (usually a title or abstract) per line, in a random order. The file had approximately 24 million lines.

### Neural network

The neural network system consisted of two neural networks––the ‘pre-training’ network and the ‘recognition’ network–with some components shared by both networks, and other components being used by only one network or the other. The ‘pre-training’ network performed a language modelling task, and was trained using unlabelled data, with the aim of using the trained weights in the shared layers as a starting point for the ‘recognition’ network, thus aiming to achieve transfer learning.

The training procedure consisted of a series of epochs, the first five of which were divided into two phases––one (phase 1) to train the ‘pre-training’ network, one (phase 2) to train the ‘recognition’ network. All subsequent epochs after the fifth omitted phase 1 and ran phase 2 only. At the end of each epoch, the system was evaluated using the development abstracts and an answer file was produced using the test abstracts. The epoch that gave the best *F* score in the evaluation phase––in the run submitted to the BioCreative challenge, the 33rd epoch––was selected and the answer file from that was submitted for official evaluation.

Each epoch of phase 1 was divided into 25 sub-epochs. In each sub-epoch, 12 000 lines of the PubMed file were read in, tokenized, and sorted into batches of 32 lines each, grouping the smallest 32 lines (by number of tokens) into one batch, the next smallest 32 into another batch, etc. Within each batch, lines that are shorter (in terms of number of tokens) than the maximum length were padded with special padding tokens. The system was trained on the batches in a random order.

For each line, a token sequence was generated, consisting of an integer representing the index of each token in a token dictionary, with a special value for unknown tokens. From this a ‘substituted’ sequence––where each token has a 0.5 chance of being replaced by a token randomly sampled from the lines read in that sub-epoch––was generated. These randomly sampled tokens act as ‘negative samples’, and the network as a whole attempts to tell whether a token in the substituted sequence is a negative sample or not.

The inputs to the ‘pre-training’ network consisted of the token sequence (input **i1**), the ‘substituted’ sequence shifted one token to the right (input **i2**; starting with padding) and the substituted sequence shifted one token to the left (ending with padding; input **i3**). There were two outputs (**d2** and **d4**), one for each of the substituted shifted sequences, consisting of a sequence of numbers––1 if the token in the substituted sequence is from the original sequence, 0 if it was randomly selected (i.e. a negative sample).

**Figure 1.**
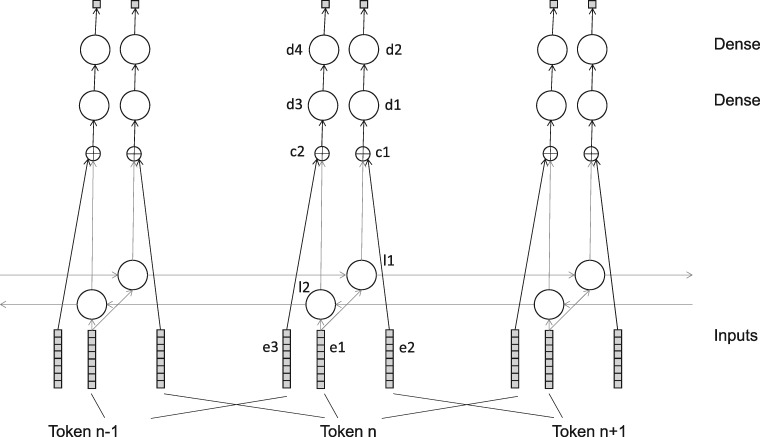
Pre-training network.

The network consisted of various layers, as shown in Table 1 and Figure 1. In all cases the number of output neurons is per token. The three embedding layers all shared the same embedding tensor. All LSTM layers were trained with a dropout and recurrent_dropout parameter of 0.5, and with return_sequences set to True.

**Table 1.** Layers in pre-training network

Layer	Type	Input(s)	Number of output neurons	Notes
**e1**	Embedding	**i1**	300	
**e2**	Embedding	**i2**	300	
**e3**	Embedding	**i3**	300	
**l1**	LSTM	**e1**	300	
**l2**	LSTM	**e1**	300	Reversed
**c1**	concatenate	**l1**, **e2**	600	
**c2**	concatenate	**l2**, **e3**	600	
**d1**	TimeDistributed Dense	**c1**	300	Activation is relu
**d2**	TimeDistributed Dense	**d1**	1	Activation is sigmoid.
**d3**	TimeDistributed Dense	**c2**	300	Activation is relu
**d4**	TimeDistributed Dense	**d3**	1	Activation is sigmoid.

In this network, **e1** represents the source sequence, **l1** represents the context to the left of a possible negative sample (in **e2**) and **l2** represents the context to the right. Layers **d1** and **d2** compare the possible negative sample to the leftward context, attempting to tell if the sample is plausible in context or not––likewise **d3** and **d4** do the same for the rightward context.

The ‘pre-training’ network was trained using RMSProp optimizer, with the binary cross-entropy loss function.

In the second phase, chemical-protein relations were detected and classified. Each epoch consisted of a single pass through the training corpus to train the network, a single pass through the development corpus to evaluate the current state of the system and a single pass through the test corpus to generate an answer file for submission.

In each pass, for each abstract, all possible chemical-protein pairs were found. Those pairs where the first token of the first entity was 60 or fewer tokens from the last token of the last entity were selected. A subsequence of tokens from the abstract was then taken, starting from 5 tokens before the first entity to 5 tokens after the last entity. The tokens for the chemical entity were replaced with ‘$CHEMICAL’ and those for the protein entity were replaced with ‘$PROTEIN’––those appearing in both entities were replaces with ‘$BOTH’. The token sequence was then converted to an integer sequence, in the same manner as the pre-training sequences were processed. Additional input sequences for each pair were also generated, consisting of an array of binary features for each token in the subsequence. One input sequence (input **i4**) consists of information about the entities in the abstract, regardless of whether they participated in the relation in question––these were features to say whether the token is in, at the start of, at the end of, overlapping the start of or overlapping the end of any chemical or protein entity. Another input sequence (input **i5**) consists of binary features to say whether the token is a part of the protein entity in question, and whether the token is a part of the chemical entity in question.

The output for the network (**d5**) was an array of 6 binary features, encoding whether and which relation exists between the two entities. The argmax of these six outputs was selected as the final output.

**Figure 2.**
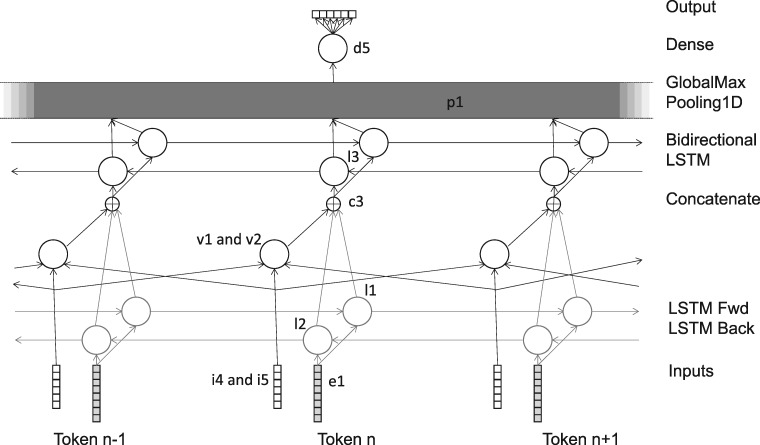
Recognition network.

The network consisted of various layers, as shown in Table 2 and Figure 2. The number of output layers is per token, except for layers **p1** and **d5**, where it is the total number overall. The layers **e1**, **l1** and **l2** are shared with the pre-training network. Again, all LSTM layers were trained with a dropout and recurrent_dropout parameter of 0.5, and with return_sequences set to True.

**Table 2.** Layers in recognition network

Layer	Type	Input(s)	Number of output neurons	Notes
**e1**	Embedding	**i1**	300	
**l1**	LSTM	**e1**	300	
**l2**	LSTM	**e1**	300	Reversed
**v1**	Conv1D	**i4**	48	Width = 3, activation is relu
**v2**	Conv1D	**i5**	6	Width = 3, activation is relu
**c3**	concatenate	**l1**, **l2**, **v1**, **v2**	652	
**l3**	Bidirectional LSTM	**c3**	128 per direction, total 256	
**p1**	GlobalMaxPooling1D	**l3**	256	
**d5**	Dense	**p1**	6	Activation is softmax

The network was trained using RMSProp, with the mean squared error loss function. During training, the candidate relationships were grouped into batches by length, if necessary padding the sequences to make the length of all the sequences in a batch uniform. The batches were then used for training in a random order.

None of the layers trained during the pre-training procedure were locked during training to the recognition network; this training was allowed to fine-tune the whole system.

On our hardware [Intel(R) Xeon(R) CPU E5-2698 v4 @ 2.20 GHz, no GPU] the time to train one epoch of Phase I pre-training was approximately 4 h, whereas an epoch Phase II training typically took 17 min.

### Additional experiments

Five batches of additional experiments were run to attempt to gain further understanding of the system.

1. *Different levels of pre-training* (Table 6)*.* The first experiment of three runs worked by successively disabling parts of the system, or replacing them with simpler alternatives. One run (‘No Phase 1’) omitted the phase 1 from training. The second (‘Public Embeddings’) also omitted phase 1, and also used the publicly available pre-trained GloVe embeddings instead of the specialized ones we had compiled ourselves. The third (‘Random’) also omitted phase 1, but using randomly-initialized embeddings instead of pre-trained ones. On the No Phase 1 run, the best epoch was the 15th epoch, on the Public Embeddings run the best epoch was the 26th epoch and on the Random run, the best epoch was the 17th epoch.
2. *Network topologies* (Table 7). A second experiment examined the importance or otherwise of using a bidirectional LSTM as layer **l3** followed by GlobalMaxPooling layer **p1**. One (‘Unidirectional, 128 outputs, final state’) used a unidirectional LSTM with 128 outputs (the same as the number of outputs per direction in the original **l3**) in place of **l3**, with the output from the final time step going directly to **d5**, with **p1** being removed. Another (‘Unidirectional, 256 outputs, final state’) used 256 outputs––the same number of outputs as the total number of outputs in the original **l3**. A third (‘Unidirectional, 128 outputs, via GlobalMaxPooling’) fed the output from the unidirectional LSTM at each token to **p1**. A fourth (‘Conv1D’) replaced **l3** with a 1D convolutional layer, with 128 outputs per token, a width of 3 and a relu activation layer. All of these were run without Phase I pre-training. A fifth (‘Conv1D with Phase I’) was as the forth but with Phase I pre-traning.
3. *Level of pre-training data* (Table 8). A third experiment used differing amounts of pre-training data. Whereas full pre-training used 25 sub-epochs per epoch––i.e. 1.5 million lines in total, the runs here used 5, 10, 15 and 20 sub-epochs per epoch (i.e. 0.3, 0.6, 0.9 and 1.2 million lines).
4. *Precision/Recall rebalancing* (Table 9). A fourth experiment repeated runs from the previous experiments. We were not using a fixed seed for the random number generator, so these experiments explore the impact of using different random initializations. Also, the experiments explored methods of varying the balance between precision and recall. The previous approach was to select the argmax of all six outputs, treating the five classes of positive outputs and the negative ‘NONE’ output the same. In this approach, a candidate positive output is selected as the argmax of the five positive outputs––we call this *x* and the value of this output *o_x_.* The negative output we call *o_0_.* We then calculate one of three values: either *a* = *o_x_*, *b* = *o_x_*–*o_0_*, or *c* = *o_x_*/*o_0_*_._ If the value is above a threshold, the candidate positive is accepted as a positive result, otherwise it is rejected. For each epoch, the threshold value and choice of *a*, *b* or *c* is determined by finding the combination of threshold value and formula that maximizes the *F* score on the development data.
5. *Development vs test set* (Table 10). The final experiment used 15 sub-epochs of pre-training per epoch, and was evaluated on the test as well as the development corpus, with and without thresholding, in order to test whether improvements made in previous experiments carried over to the test set.

Results of these experiments are discussed in the next section.

## Results and discussion

Table 3 shows the results from the task:

**Table 3.** Results

Corpus	Precision (%)	Recall (%)	*F* (%)
Development	56.52	70.42	62.71
Test	56.10	67.84	61.41

The *F* of <63% indicates that there is considerable room for improvement on this task. This is the first time that BioCreative has tackled a chemical-protein interaction task––however, in the past it has considered chemical-disease relations (getting a maximum *F* score of 57.03%; 20) and protein–protein interactions (getting a maximum *F* of 55%; 21). These relationship-mining tasks appear to be harder than named entity extraction tasks, where *F* scores in excess of 80% are routine and *F* scores above 90% are not unknown (22). There appears to have been a slight loss of performance between the development and test––it is possible that this is because the gains from selecting the best epoch did not generalize well.

The system with the highest *F* score, by Peng *et al.* (23) reported an *F* of 64.10% (2.69 percentage points ahead of ours), using an ensemble of three systems, including an LSTM-based system. Our team was the second-placed team on *F* score (1), achieved the highest recall and seventh (out of 13) for precision.

Table 4 shows a confusion matrix for the development data, and Table 5 shows a breakdown of the results on the development data by relationship class.

**Table 4.** Confusion matrix for development data

Actual	Predicted					
	*NONE*	*CPR: 3*	*CPR: 4*	*CPR: 5*	*CPR: 6*	*CPR: 9*
***NONE***	26196	214	413	75	76	351
***CPR: 3***	163	287	82	4	4	9
***CPR: 4***	159	24	896	0	5	6
***CPR: 5***	23	0	0	89	4	0
***CPR: 6***	28	1	7	3	160	0
***CPR: 9***	166	4	16	0	2	258

**Table 5.** Development data results by relationship class

Class	Precision (%)	Recall (%)	*F* (%)
***CPR: 3***	54.16	52.28	53.20
***CPR: 4***	63.37	82.20	71.57
***CPR: 5***	52.04	76.72	62.02
***CPR: 6***	63.75	80.40	71.11
***CPR: 9***	41.35	57.85	48.22

The major source of error seems to be non-relations being mistaken for relations and vice versa. There is something of a problem with upregulation (CPR: 4) being mistaken for downregulation (CPR: 3) but the bigger problem for these classes is confusion with NONE.

There is considerable variation in how well these entities are recognized––CPR: 4 (downregulator/inhibitor) and CPR: 6 (antagonist) are well-recognized, CPR: 3 (upregulator) and CPR: 9 (substrate/product) are poorly recognized. The *F* scores do not appear to be correlated with the number of mentions in the corpus. Two other participants in BioCreative VI also studied the variation in how well the entities were recognized. Tripodi *et al.* (24) found a different pattern in results to ours, whereas Liu *et al.* (25) found a similar pattern (our highest-scoring entity type was their highest scoring entity type, and so on for the second and third highest scoring entity types). The results of Tripodi *et al.* may not be directly comparable to ours in this case, as they came from evaluation on 20% of the training data, whereas Liu *et al.* and ourselves used the development data.

Table 6 shows the results of re-running the system, progressively disabling parts of the system that make use of unlabelled data. The Phase 1 training of the lower LSTMs is shown to improve performance by 2.7 percentage points. Using specialized embeddings improves performance by 0.7 percentage points over the off-the-shelf embeddings, and the off-the-shelf embeddings give 11.5 percentage points over random initialization.

**Table 6.** Results on development

Run	Precision (%)	Recall (%)	*F* (%)
Full	56.52	70.42	62.71
No Phase 1	62.97	57.25	59.97
PublicEmbeddings	61.69	56.96	59.23
Random	45.05	50.66	47.70

Table 7 shows the effects of replacing the second bidirectional LSTM layer (**l3**) with various alternatives, along with the ‘Full’ and ‘No Phase I’ results for reference. The GlobalMaxPooling layer appears to be important; collecting the output at the end of a unidirectional LSTM gives worse results. However, without Phase I pre-training, the Bidirectional LSTM layer is not necessarily the best––we obtained better results with a unidirectional LSTM layer, and with a 1 D Convolutional layer. However the system with the 1 D Convolutional layer did not appear to benefit from Phase I pre-training, instead performing worse; recall was improved, but at the expense of precision.

**Table 7.** Results on development

Run	Precision (%)	Recall (%)	*F* (%)	Best Epoch
Full	56.52	**70.42**	62.71	33
No Phase I	62.97	57.25	59.97	15
Unidirectional, 128 outputs, final state	56.49	58.96	57.69	10
Unidirectional, 256 outputs, final state	50.71	62.33	55.93	24
Unidirectional, 128 outputs, via Global MaxPooling	61.70	60.88	61.28	6
Conv1D	**67.05**	60.04	**63.35**	28
Conv1D with Phase I	56.39	64.13	60.01	48

Numbers in boldface represent best results.

These experiments are not a comprehensive exploration of all possible variations on the network architecture. The results indicate that there is potentially some scope for improvement with further experimentation.

Table 8 shows the results of adjusting the amount of data used in Phase I pre-training. As more data is added it increases recall at the expense of precision, at first improving and then worsening the *F* score. It is not clear why the transfer learning improves recall specifically, however this effect may account for our system achieving the highest recall in the challenge.

**Table 8.** Results on development

Sub-Epochs	Precision (%)	Recall (%)	*F* (%)	Best Epoch
0	**62.97**	57.25	59.97	15
5	61.37	64.67	62.97	19
10	61.69	64.88	63.24	12
15	59.38	68.29	**63.53**	36
20	57.06	69.92	62.83	38
25	56.52	**70.42**	62.71	33

Numbers in boldface represent best results.

We were surprised by this pattern of improvement then worsening. With supervised learning, outside of the context of transfer learning, using more training data than is necessary may use more computer time than necessary, but if all of the data is sampled evenly from the same source, it is commonly believed that it is unlikely to substantially negatively affect the quality of the results even if it does not have a beneficial effect. With transfer learning, there is the known phenomenon of ‘negative transfer’, where pre-training on one task can negatively affect performance on other tasks. Two recent reviews of transfer learning (26, 27) have both noted the area of negative transfer has not been widely researched. They discuss various strategies for preventing negative transfer. However, there appears to be no mention of the phenomenon whereby a certain amount of data achieves positive transfer and additional data beyond that removes some of the benefit of that positive transfer, and no mention of a strategy of maximizing the positive transfer merely by restricting the amount of transfer learning data used.

Considering that this phenomenon is new to us, we needed to rule out some of the ways this appearance of worsening could be illusory. One concern is that we had not used a fixed seed for the random number generator, so there was some amount of run-to-run variability. A second is that *F* scores tend to be higher when precision and recall are roughly equal, and using large amounts of pre-training data appears to emphasize recall. Re-running the key experiments using a thresholding approach that balances precision and recall allows us to address both questions.

Table 9 shows the results of these experiments. Comparison with previous results shows some run-to-run variability––the 25, 15 and 0 sub-epoch experiments showed results improved by 1.35, 1.08 and 0.18 percentage points, and the Conv1D experiment was worsened by 1.79. This level of variability raises some questions about how much information can be gathered using these experiments, and also suggests a strategy for improving overall performance––run several runs with the same hyperparameters but different random seeds, and select the best one. However, the results in Table 9 for the 25, 15 and 0 sub-epochs do reproduce the trend seen in Table 8, reinforcing the conclusion that there is an optimal amount of data to use in Phase I and using too much can be detrimental. The reduced performance of the Conv1D experiment suggests that its earlier good performance was at least somewhat fortuitous.

**Table 9.** Results on development

Run	Precision (%)	Recall (%)	*F* (%)	*Best Epoch*
25 Sub-Epochs	71.08	59.92	65.02	26
	58.22	68.79	63.06	24
15 Sub-Epochs	68.87	62.58	65.58	15
	63.05	66.25	64.61	15
No Phase I	63.20	59.45	61.27	22
	56.26	64.63	60.15	18
Conv1D, No Phase I	65.86	59.50	62.52	19
	55.94	68.42	61.56	19

For each run, the upper row is with thresholding, the lower is without.

The use of thresholding techniques to boost *F* score gave boosts of 1.96, 0.97, 1.12 and 0.96 percentage points for the 25, 15 and 0 sub-epoch and Conv1D experiments, respectively. The effect is more dramatic for 25 than for 15 sub-epochs, showing that some of the advantage of 15 over 25 came from 15 giving a more even precision/recall balance. It is also clear that the advantage of Phase I is not a simple matter of finding a better precision/recall balance. Of the six runs in Table 8, and the top three in Table 9, every run that involved Phase I pre-training outperformed every run that did not––regardless of whether thresholding was used or not.

There is a final concern that the techniques of selecting the best threshold may amount to overfitting to the development data, and possibly that the use of 15 sub-epochs rather than 25 might be similar. To make a final check, we re-ran the system one final time, using 15 sub-epochs. For each epoch, we found the threshold and thresholding method that gave the maximum *F* score on the development data. That epoch’s model, threshold and method were then evaluated on the test set using the official evaluation tool. We also tried not using thresholding (on the same run), selecting the epoch that maximized the *F* score on the development data and using the model from that epoch for evaluation with the test data.

The results in Table 10 show that the improvements from reducing the number of sub-epochs of Phase I pre-training can be reproduced in the test set. The gain in the test set performance associated with thresholding appears to be slight.

**Table 10.** Results on development and test

Run	Precision (%)	Recall (%)	*F* (%)	Best Epoch
Development, no thresholding	**63.33**	65.42	64.36	19
Test, no thresholding	62.56	62.52	62.54	
Development, thresholding	63.87	67.17	64.91	34
Test, thresholding	62.97	62.20	62.58	

Numbers in boldface represent best results.

**Figure 3.**
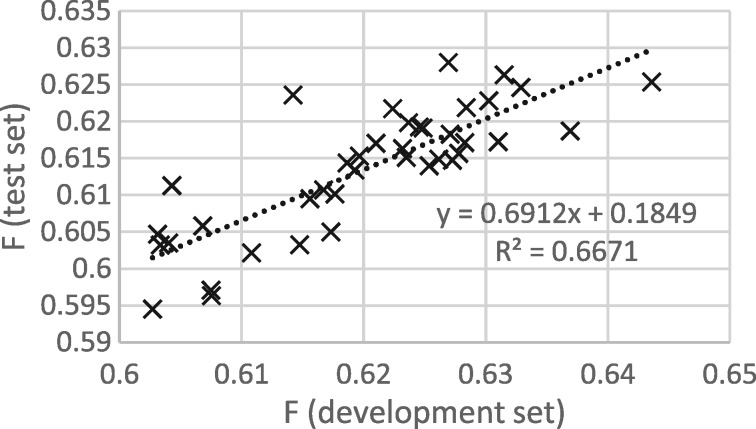
*F* scores for development and test sets from epochs 12 to 50, with thresholding.

**Figure 4.**
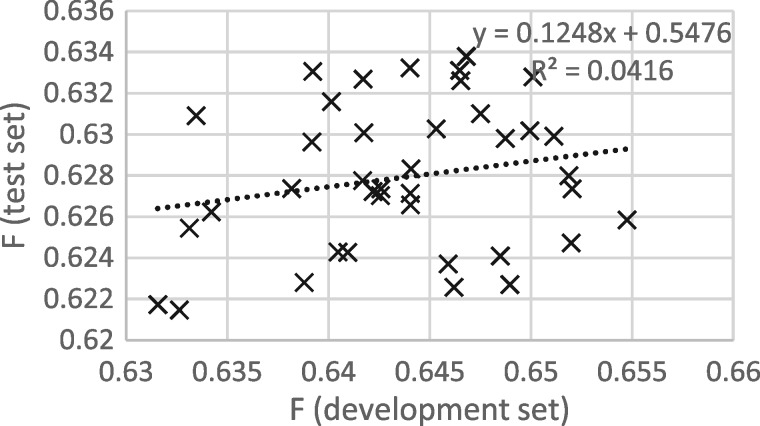
*F* scores for development and test sets from epochs 12 to 50, with thresholding.

Figures 3 and 4 show that the procedure of selecting the epoch that gives the best performance on the development set gives good results when thresholding is not applied, but not when thresholding is applied. It appears that some of the epoch-to-epoch variability is due to the precision/recall balance, that balance carries over from the development to the test set and that thresholding removes that variability, greatly reducing the usefulness of epoch selection.

## Conclusion

Methods based on ‘deep learning’ RNNs can be used to detect relationships between chemicals and protein, with results comparable with those observed in other biomedical relationship extraction tasks. The deep learning structure allows the use of large amounts of unlabelled text to boost performance, especially via the use of pre-trained word embeddings. It is notable that our system makes minimal use of external natural language processing resources beyond the unlabelled data––it does use a chemistry-aware tokenizer, but makes no use of sentence splitters, POS taggers, stemmers, parsers, ontologies or other such resources.

Transfer learning and specialized embeddings provide methods for learning from large amounts of data that are not directly linked to a specific task. We have found that using transfer learning from PubMed gave a 2.7 percentage point improvement, and specialized embeddings gave a 0.7 percentage point improvement. The techniques and results discussed in this paper show that there is scope for considerable more experimentation in applying transfer learning and associated methods to improve the extraction of knowledge about biological interactions from literature.

One odd aspect of the transfer learning is that the best performance is not obtained by using as much transfer learning data as possible, but by experimenting to find the optimal amount––additional data beyond that amount makes things worse. The reason for this is not yet clear to us. Conceivably, finding and alleviating the cause of this worsening could lead to further improvements on this task, and provide insight into how transfer learning could be best used in other natural language processing tasks. Other potential areas for improvement include exploring variations in network architecture, and improving the handling of out-of-vocabulary words, possibly by re-using approaches from NER, POS tagging or similar tasks.

The source code for our system is available on-line, as a part of the distribution for ChemListem, at https://bitbucket.org/rscapplications/chemlistem.
